# Dietary Protein and Transitions Between Frailty States and to Death in Advanced Age: LiLACS NZ

**DOI:** 10.1093/geroni/igaa057.772

**Published:** 2020-12-16

**Authors:** Ruth Teh, Ngaire Kerse, Nuno Mendonca, Oliver Menzies, Tom Hill, Carol Jagger

**Affiliations:** 1 University of Auckland, Auckland, Auckland, New Zealand; 2 Newcastle University Institute for Ageing, Newcastle upon Tyne, England, United Kingdom; 3 Auckland District Health Board, Auckland, Auckland, New Zealand; 4 Newcastle University, Newcastle upon Tyne, England, United Kingdom

## Abstract

Modifiable factors delaying frailty progression are important in demographic ageing; health disparities for indigenous people require specific strategies. Does dietary protein intake impact transitions in frailty in Maori (indigenous) and non-Maori aged 80+ in New Zealand? LiLACS NZ is population based longitudinal cohorts of Maori aged 80-90 years and non-Maori aged 85 years, followed yearly to 5 years follow up. At 12 months follow up 459 participants contributed nutrient intakes from 24hr multiple pass recall dietary intake. Frailty states were derived from Fried frailty criteria. Mortality was established through National Health Index matching. Multistate modelling investigated the contribution of protein intake to transitions in frailty states and death using models of increasing complexity: 1)age, ethnicity; 2)sex; 3)disease counts; and 4)energy intake. Over 60% of the sample were prefrail throughout the study; disease burden differentiated frailty state. Of a total of 1269 transitions, 692 remained the same; the models used 549 transitions; 44% from robust to pre-frail or pre-frail to frail. Those recovering from pre-frail to robust had lower disease burden and higher nutritional intake. Those with higher protein intake were less likely to transition from robust to prefrail (model 4: per 1g/kg bodyweight/d: HR: 0.28, 95%CI: 0.08-0.91) and from pre-frail to robust (0.24 0.06-0.93). Increased protein intake was associated with increased transition from frailty to mortality but was moderated by energy intake. Greater protein intake in octogenarians in NZ was associated with both better and worse outcomes. Total energy intake tended to moderate negative outcomes.

